# Alleviation of Chlorpyrifos Toxicity in Maize (*Zea mays* L.) by Reducing Its Uptake and Oxidative Stress in Response to Soil-Applied Compost and Biochar Amendments

**DOI:** 10.3390/plants10102170

**Published:** 2021-10-14

**Authors:** Humera Aziz, Ghulam Murtaza, Muhammad Hamzah Saleem, Shafaqat Ali, Muhammad Rizwan, Umair Riaz, Abdullah Niaz, Muyassar H. Abualreesh, Aishah Alatawi

**Affiliations:** 1 Department of Environmental Sciences and Engineering, Government College University, Faisalabad 38040, Pakistan; humeraaziz.uaf@gmail.com (H.A.); mrazi1532@yahoo.com (M.R.); 2 Institute of Soil and Environmental Sciences, University of Agriculture, Faisalabad 38000, Pakistan; 3 College of Plant Science and Technology, Huazhong Agricultural University, Wuhan 430070, China; saleemhamza312@webmail.hzau.edu.cn; 4 Department of Biological Sciences and Technology, China Medical University, Taichung 40402, Taiwan; 5 Soil, Water and Fertilizer Testing Laboratory for Research, Bahawalpur 63100, Pakistan; riaz44@gmail.com; 6 Pesticide Residue Laboratory Kala Shah Kaku, Sheikhupura 39350, Pakistan; Niaz55@gmail.com; 7 Department of Marine Biology, Faculty of Marine Sciences, King Abdualaziz University, Jeddah 21589, Saudi Arabia; Mabulreesh1@kau.edu.sa; 8 Biology Department, Faculty of Science, Tabuk University, Tabuk 71421, Saudi Arabia; Amm.alatawi@ut.edu.sa

**Keywords:** chlorinated organophosphates, soil pollution, biochar, compost, remediation, food safety

## Abstract

Chlorpyrifos (CP) is a pesticide used extensively in agricultural crops. Residual CP has been found in a variety of soils, vegetables and fruits indicating a serious danger to humans. Therefore, it is necessary to restrict its entry into agricultural products for food safety. A wire-house pot experiment was conducted with maize plants in biochar- and compost-amended soil (at 0.25% and 0.50%, respectively, in weight-by-weight composition) contaminated with 100 and 200 mg kg^−1^ of CP, respectively. Results indicated toxicity at both CP levels (with 84% growth reduction) at CP 200 mg kg^−1^. However, application of compost and biochar at the 0.50% level improved the fresh weight (2.8- and 4-fold, respectively). Stimulated superoxide dismutase (SOD) and peroxidase (POX) activities and depressed catalase (CAT) activity were recorded in response to CP contamination and were significantly recovered by the amendments. Both amendments significantly decreased the CP phytoavailability. With biochar, 91% and 76% reduction in the CP concentration in maize shoots and with compost 72% and 68% reduction was recorded, at a 0.50% level in 100 and 200 mg kg^−1^ contaminated treatments respectively. Compost accelerated the CP degradation in postharvest soil. Therefore, biochar and compost amendments can effectively be used to decrease CP entry in agricultural produce by reducing its phytoavailability.

## 1. Introduction

Pesticides are widely used to increase agricultural production by controlling pests, however, they have strong potential to severely contaminate agricultural soils [1]. Organophosphorus pesticides usage has been aggravated worldwide due to their high efficacy towards killing insect pests [2]. Chlorpyrifos (CP) [O, O-Diethyl O-(3, 5, 6-trichloro-2-pyridinyl)-phosphorothioate] is a toxic chlorinated organophosphorus insecticide. Its half-life in soil ranges from 60 to 120 days [3]. Its use on a large scale contaminates various components of the environment such as soil, water and terrestrial ecosystems [4]. CP residual concentrations have been reported in ground water, soil, vegetables, edible fruits, fish and in cow meat in Pakistan [5,6,7,8], which clearly highlights the associated health risks owing to its entrance into the food chain [9]. CP applied to plants directly or mixed with soil may produce adverse effects on the environment [10]. Moreover, CP exerts drastic effects on plant growth, as indicated by seedling growth inhibition, reduction in root and shoot growth, germination energy, germination percentage chlorophyll contents and morphological traits [10,11,12,13,14].

Organic amendment application in soil is recommended as a good practice to improve soil fertility and crop productivity. The agricultural soils of Pakistan are characterized by poor organic carbon contents [15], having less than 10 mg g^−1^ soil organic carbon contents in most soils [16]. Therefore, soil incorporation with organic materials such as compost, biochar and manures, is considered a highly profitable practice to improve the organic matter status of soils [17,18,19]. Moreover, these amendments alter the physical and chemical properties of soil [20,21] which ultimately affect the fate of pesticides in soils [22]. Pesticide adsorption of biochar decreases the availability of harmful organic contaminants present in soil to organisms and restricts their transport to the receiving environment [23,24]. Supplementation of soil with activated carbon or biochar can decrease the pesticide uptake by plants [11,25]. Wheat- and rice-straw-derived biochar was found to be 2500 times more effective towards pesticide sorption when compared with soil [26]. Enhanced sorption of CP by biochar was also reported elsewhere [22]. There is a dire need to develop procedures to explore the immobilization mechanisms of CP by organic amendments. Previous studies have reported the effects of pesticide toxicity on different plants. The positive effect of biochar has been reported in previous studies by recovering antioxidant enzyme activities under pesticide pollution. However, studies regarding the effect of compost on CP toxicity in soil–plant systems and on antioxidant enzyme activities in CP-stressed maize plants are very rare. Moreover, the comparative behaviors of biochar and compost on CP uptake by maize plants and on antioxidant enzyme activities under CP toxicity in Pakistani soil conditions is lacking in the literature. Considering the above scenario, this study was designed with the following objectives: (1) to investigate the uptake of CP by maize plants, (2) to evaluate and compare the potential of two types of organic amendments (biochar and compost) at two different levels in reducing the bioavailability of CP to maize plants, and (3) to elucidate the behavior of antioxidant enzymes in CP-contaminated soil in response to soil-applied biochar and compost.

## 2. Results and Discussion

### 2.1. Soil and Amendments Characteristics

The soil used for experiment had a sandy clay loam texture, ECw (1:10) 3.21 dS m^−1^, pH_w_ (1:10) 7.44, with low organic carbon contents (0.87%). The CaCO_3_ contents of soil were 4.91%. The biochar contained high total organic carbon contents, 43.8%, compared to compost. Significantly high BET specific surface area and lower pore width was recorded in biochar when compared with compost (Table 1). The maximum (107.5 cmol_c_ kg^−1^) CEC was found in compost, while the minimum (5.2 cmol_c_ kg^−1^) was found in soil. The Fe, Mn and Zn contents of soil were 5.5, 0.51 and 0.91 mg kg^−1^, respectively. The compost exhibited high contents of Fe and Zn (755, 130 mg kg^−1^) compared to biochar (154, 78 mg kg^−1^). The total N, available phosphorous and extractable K contents were found in the order of compost > biochar > soil.

### 2.2. Plant Growth

**Figure 1:**
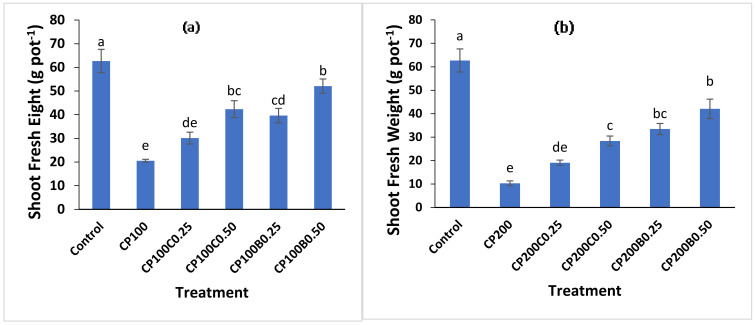
Fresh weight of maize shoot as affected by the CP toxicity (**a**) at 100 mg kg^−1^ (**b**) at 200 mg kg^−1^ of soil and the alleviating effect of compost and biochar application. Bars sharing different letters are statistically different from each other and vice versa at *p* < (0.05).

**Figure 2:**
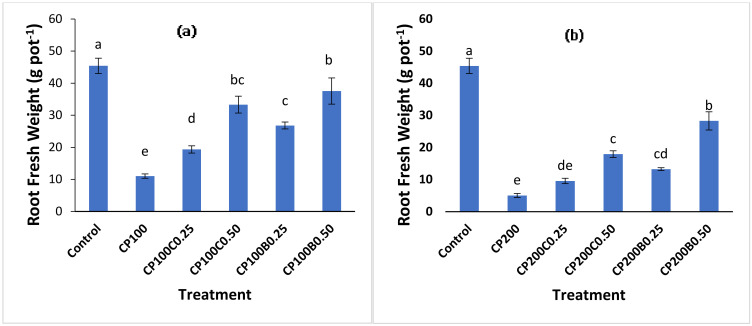
Fresh weight of maize roots as affected by the CP toxicity (**a**) at 100 mg kg^−1^ (**b**) at 200 mg kg^−1^ of soil and the alleviating effect of compost and biochar application. Bars sharing different letters are statistically different from each other and vice versa at *p* < (0.05).

CP significantly (*p* < 0.05) reduced the shoot (Figure 1a,b) and root fresh (Figure 2a,b) weights of maize plants. The addition of biochar and compost alleviated the damaging effects of CP on shoot fresh weight and increased the shoot fresh weight compared to those plants where only CP was applied. The plants grown with CP_100_ and CP_200_ (100 and 200 mg kg^−1^ of CP, respectively) produced 67% and 84% less shoot fresh weights, respectively, compared to control (CP_0_B_0_C_0_) plants. A decrease in maize growth due to CP toxicity can be attributed to the inhibition of the activity of 4-hydroxyl phenyl pyruvate dioxygenase (HPPD), which is needed for growth and development of meristematic tissue [27]. However, supplementation with compost and biochar recovered this damaging effect at both levels but more pronouncedly at the 0.50% level of both amendments. Moreover, biochar was found to be more effective in restoring the maize biomass in all contaminated treatments compared to compost-amended treatments. The highest shoot fresh weight was recorded with CP_100_B_0.50_ (52.07 g pot^−1^) a significant (*p* < 0.05) increment of 154% compared to CP_100_, while with compost this increment was 107% with CP_100_C_0.50_ compared to CP_100_. At CP200 level, CP_200_B_0.50_ and CP_200_C_0.50_ showed 175% and 307% increments in shoot fresh weights compared to unamended CP_200_. The root fresh weight ranged between 5.05 and 45.4 g pot^−1^. The minimum root fresh weight (5.05 g pot^−1^) was recorded with CP_200_ (a decrease of 89%) compared to the control plants. The 0.50% level of both amendments (compost and biochar) in combination with CP_100_ recovered this reduction in root fresh biomass significantly and exhibited 4- and 5-fold increase in root fresh weight, respectively, compared to the treatments with CP_100_ alone. The inhibiting effect of CP (75 and 100 mg kg^−1^) on the seedling growth of two grass species has been previously reported [28]. The suppression of shoot and root biomass of plants by CP toxicity and significant recovery of this reduction by biochar supplementation in soil has also been reported by [11] and [29]. Reduction in plant growth in response to CP toxicity was also reported by [14]. The incorporation of organic amendments in contaminated soil improves plant growth by reducing plant access to pesticide residues in soil [30], minimizing negative impacts on soil enzyme activities and soil microbial population due to increased soil organic matter [31] by directly applying nutrients [11] and improving physical and biological properties of soil [32].

### 2.3. Antioxidant Enzyme Activities of Maize Shoots

**Figure 3:**
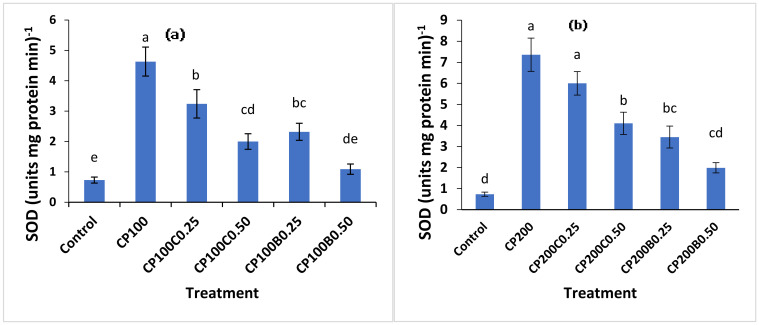
Variation in SOD activities of maize plants grown in CP contaminated soil (**a**) at 100 mg kg^−1^ (**b**) at 200 mg kg^−1^ of soil and the effect of compost and biochar applied. Bars sharing similar letters are not statistically different from one another and vice versa at *p* < 0.05.

The enzymatic antioxidant system is most important strategy for plants to respond to environmental stress [14]. In the case of insecticide toxicity, plants prevent oxidative damage to their cells to tolerate this stress. Most often, this toxicity boosts the activities of SOD and POX, which are indicators of the degree of stress as well as the ability of stress tolerance [14]. The effect of CP on antioxidant enzyme SOD activity of maize shoots in amended and unamended soils is shown in (Figure 3a,b). The SOD activity of maize plants was significantly (*p* < 0.05) promoted in CP-stressed plants compared to untreated control plants. The maize shoots with CP_100_ and CP_200_ showed 5- and 8-fold increments in SOD activity (U (mg^−1^ protein min^−1^) compared with the control. The compost- and biochar-supplemented plants showed lower SOD activities compared with unamended CP-contaminated plants. The restoration of SOD activity was more evident at the 0.50% level of both amendments. However, biochar-amended treatments showed significantly (*p* < 0.05) less SOD activities in all contaminated treatments compared to compost-amended treatments. Among the amended treatments, the CP_100_C_0.50_, CP_100_B_0.50_, CP_200_C_0.50_ and CP_200_B_0.50_ showed 45%, 70%, 42% and 75% less SOD activity compared with unamended CP_100_ and CP_200_, respectively.

**Figure 4:**
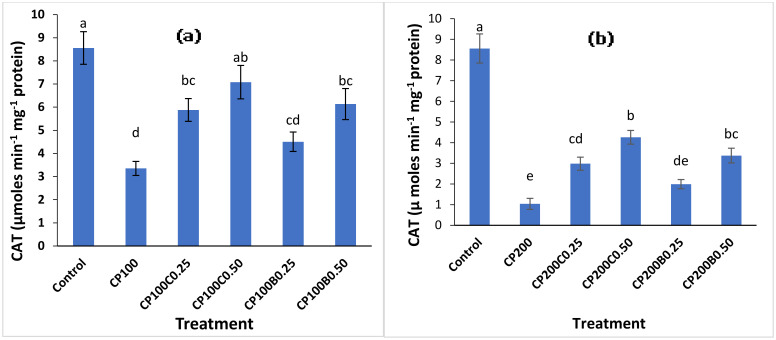
Variation in CAT activities of maize plants grown in CP-contaminated soil (**a**) at 100 mg kg^−1^ (**b**) at 200 mg kg^−1^ of soil and the effect of compost and biochar applied. Bars sharing similar letters are not statistically different from one another and vice versa at *p* < (0.05).

In contrast to SOD, the CAT activity of maize shoots showed opposite behavior in response to CP toxicity (Figure 4a,b). CP significantly (*p* < 0.05) depressed the CAT activity of maize plants in all treatments (with and without amendment addition), except untreated control plants. The CP_100_ and CP_200_ showed 61% and 88% reductions in CAT activity compared to control plants. The CAT activity ranged between 1.04 with CP_200_ and 8.56 (µmoles Min^−1^ mg^−1^ protein) in control plants. The application of both amendments recovered the reduction in CAT activity. Among the compost-amended treatments, the CP_100_C_0.25_ and CP_100_C_0.50_ caused 76% and 111% increments in CAT activity, respectively, compared with unamended CP_100_, while for biochar these increments were 34% and 83% with CP_100_B_0.25_ and CP_100_B_0.50_, respectively. The combinations of compost and biochar with CP_200_ resulted in 2- and 4-fold increments with CP_200_C_0.25_ and CP_200_C_0.50_ and 1.9- and 3.2-fold increments in CAT activity with CP_200_B_0.25_ and CP_200_B_0.50_, respectively, over unamended CP_200_.

**Figure 5:**
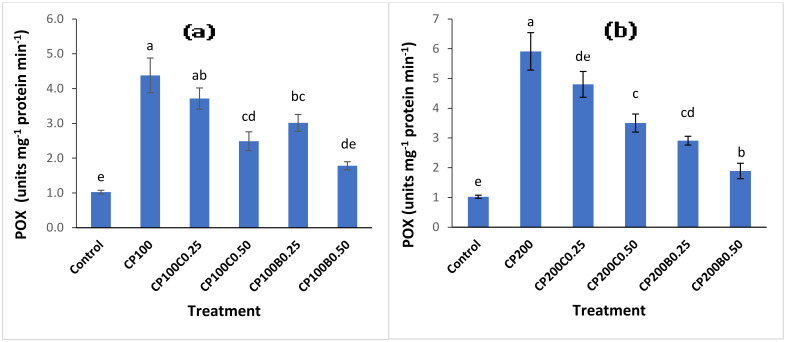
Variation in POX activities of maize plants grown in CP-contaminated soil (**a**) at 100 mg kg^−1^ (**b**) at 200 mg kg^−1^ of soil and the effect of compost and biochar applied. Bars sharing similar letters are not statistically different from one another and vice versa at *p* < 0.05.

CP application significantly (*p* < 0.05) stimulated POX activity. The CP_100_ and CP_200_ showed 4- and 6-fold increments in POX activity compared with untreated control plants (Figure 5a,b). The addition of both amendments at the 0.50% level in CP-contaminated treatments resulted in significantly (*p* < 0.05) decreased activity of POX compared to the treatments where only CP was applied. At the CP_100_ level, 43% and 59% reductions in POX activity were recorded with CP_100_C_0.50_ and CP_100_B_0.50_, respectively, while at the CP_200_ level, 39% and 68% reductions in peroxidase activity were observed with CP_200_C_0.50_ and CP_200_B_0.50_, respectively, compared with unamended CP_100_ andCP_200_.

One of the main toxic effects of CP is reactive oxygen species (ROS)-induced damage to the plants. To mitigate and repair the damage caused by these ROS, plants have evolved complex antioxidant systems. The stimulation of SOD and POX activities under CP toxicity confirms the large amount of O^2−^ production [33,34]. The CP-stressed maize plants showed significant enhancements in SOD and POX activities, which are the indicators of CP toxicity in this study. Some previous studies [14,35] reported increased SOD and POX activities upon CP stress in mung bean and wheat plants, which is consistent with our findings with maize. SOD and POX play an important role in the dismutation of free radicals by the formation of H_2_O_2_. The breakdown of H_2_O_2_ and lignin biosynthesis in the presence of H_2_O_2_ is participated by POX under pesticide toxicity [35]. Contrary to SOD and POX activities, we found depressed CAT activity under CP toxicity. The decreased CAT activity in *Glycine max* L. under insecticide stress [36] and under herbicide stress in wheat root has been reported [37]. The reduction in CAT activity may be attributed to changes in enzyme structure as a result of binding nonessential metals, degradation of enzymes by peroxisomal protease causing enzyme inactivation and changes in the assembly of CAT subunits [34,38,39]. The recovery of enzyme activities in response to organic amendments may be due to improved plant growth, water contents and nutrients uptake in poor quality contaminated soil [40]. 

### 2.4. Chlorpyrifos Residues in Postharvest Soil

**Figure 6:**
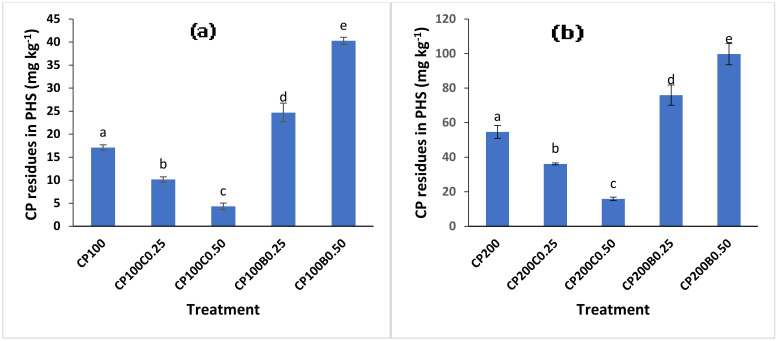
Effect of compost and biochar on CP residual concentration in postharvest soil (PHS). (**a**) at 100 mg kg^−1^ of CP (**b**) at 200 mg kg^−1^ of CP. Bars sharing similar letters have no significant difference with respect to others and vice versa at *p* < 0.05.

The residues of CP in postharvest soil are shown in (Figure 6a,b). The incorporation of biochar amendment resulted in a significantly (*p* < 0.05) reduced CP loss in soil, while compost-amended treatments enhanced the CP residue degradation in postharvest soil. In biochar-amended treatments, 45% and 136% more CP residues in postharvest were recorded with CP_100_B_0.25_ and CP_100_B_0.50_, respectively, compared to unamended CP_100_. While with the CP_200_ level, 39% and 82% increments in postharvest CP residues were recorded with CP_200_B_0.25_ and CP_200_B_0.50_, respectively, when compared with unamended CP_200_. The reduced dissipation of CP in amended soil is due to strong sorption and less desorption from organic amendments [22,41] for microbes. A decrease in CP dissipation in planted soil in response to biochar addition has been reported [11,41]. We found significantly (*p* < 0.05) less residues of CP in compost-amended postharvest soil at the end of the experiment compared with unamended as well as biochar-amended soil. At the CP100 level, a 41% and 76% lower concentration of CP was recorded with CP_100_C_0.25_ and CP_100_C_0.50_, respectively, compared with unamended CP_100_. While with CP_200_C_0.25_ and CP_200_C_0.50_ treatments, 33% and 69% less CP residues were recorded, respectively, compared with CP_200_. The increased degradation of CP was due to an enhanced microbial population introduced by the addition of organic material such as compost [42]. The microbes can utilize labile carbon provided by compost [43]. The indigenous microbes of composted material secrete some extracellular enzymes which have the ability to increase the pesticide degradation by converting their hydrophobic structures to being hydrophilic in nature [44,45]. 

### 2.5. Chlorpyrifos Concentration in Maize Plants in the Presence and Absence of Organic Amendments

**Figure 7:**
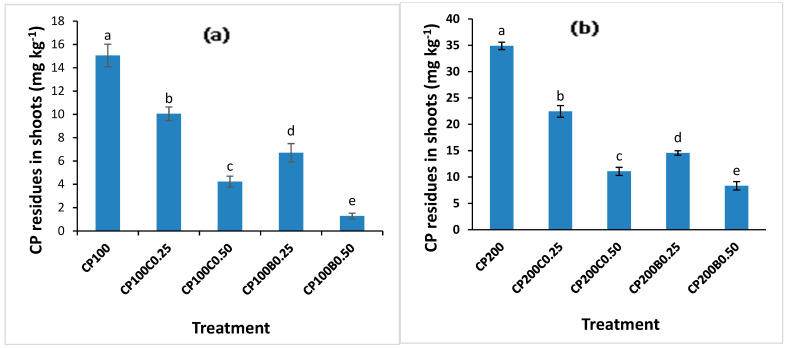
Effect of compost and biochar on CP residual concentration in shoots of maize (**a**) at 100 mg kg^−1^ of CP (**b**) at 200 mg kg^−1^ of CP. Bars sharing similar letters have no significant difference with respect to others and vice versa at *p* < 0.05.

**Figure 8:**
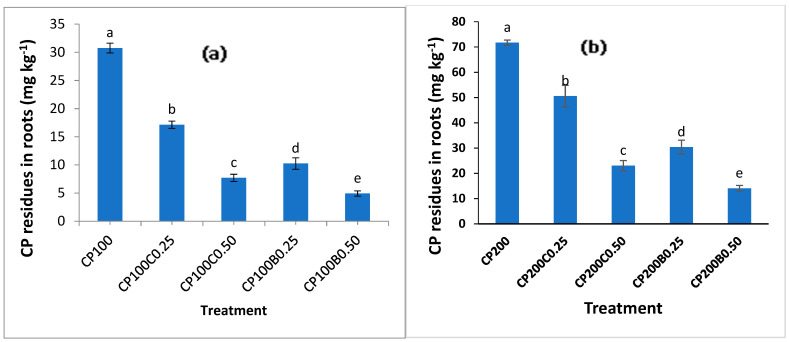
Effect of compost and biochar on CP residual concentration in roots of maize (**a**) at 100 mg kg^−1^ of CP (**b**) at 200 mg kg^−1^ of CP. Bars sharing similar letters have no significant difference with respect to others and vice versa at *p* < 0.05.

The residues of CP were determined in both shoots and roots of maize after 60 days of growth. Both CP levels significantly (*p* < 0.05) increased the CP concentration in shoots. A significant (*p* < 0.05) reduction in CP accumulation in maize shoots was recorded with both compost- and biochar-amended treatments (Figure 7a,b) compared with unamended treatments. However, for both levels of CP (CP_100_ and CP_200_), biochar-amended soil exhibited significantly less CP concentration in shoots compared with compost-amended soil. Moreover, by increasing the level of amendments from 0.25% to 0.50%, a further decrease in CP concentration in shoots was recorded. The concentration of CP in shoots was reduced from 15.06 mg kg^−1^ with CP_100_ to 1.28 mg kg^−1^ with CP_100_B_0.50_, showing 91% reduction, while at the CP_200_ level this reduction was 76% with CP_200_B_0.50_. In the case of compost-application, the reductions in CP shoot concentration were recorded as 72% and 68% with CP_100_C_0.50_ and CP_200_C_0.50,_ compared with unamended CP_100_ and CP_200_, respectively. A similar trend was found with roots (Figure 8a,b) where maximum (71.69 mg kg^−1^) CP concentration was found with CP_200_ which decreased to minimum (4.92 mg kg^−1^) with CP_100_B_0.50,_ showing a maximum decrease of 84%. In the case of compost supplementation, a 75% and 68% reduction in CP root concentration was recorded with CP_100_C_0.50_ and CP_200_C_0.50_ compared with unamended CP_100_ and CP_200_, respectively. Both amendments showed effective results in lowering uptake of CP by maize plants as shown by the greater biomass in amended treatments compared with the unamended treatments contaminated with CP. The increase in CP concentration of plants as a result of its application has been reported in wheat [46]. Organic matter added in soil provides the most important sorbent surfaces for the nonpolar pesticides having low water solubility, because phase partitioning is driven by hydrophobic interactions [46]. The mechanism behind the low bioavailability of pesticides is the sorption of pesticides on organic amendments. The microporosity and high specific surface area of biochar and a variety of functional groups provided by humic-like molecules and increased specific surface area due to humification of organic macromolecules makes them very efficient sorbent materials for a variety of organic contaminants, which in turn minimizes the risk of contaminant entrance into the food chain [47,48,49,50,51], hence reducing their toxicity. The low bioavailability of the herbicide fomesafen to maize plants in biochar-amended soil was reported previously [52]. The decreased bioavailability of CP to plants as a result of organic amendment addition in soil can be due to reduction in degradation and enhanced retention of pesticides in soil because of low bioavailability of pesticides to soil microorganisms, and the second reason is the lower uptake in plant parts which may be attributed to reduction in phytoavailability of this pesticide [11,53]. The present results confirm these findings, as low CP concentration was recorded in maize shoots and roots due to biochar and compost addition in soil. 

## 3. Materials and Methods

### 3.1. Collection and Preparation of Soil and Amendments

Soil was collected (0–30 cm depth, random method) from the farm area of Village No.132/GB in the district of Faisalabad, Pakistan. The soil was passed through a 2 mm sieve after air drying and pulverization. The soil is moderately calcareous, canal-water irrigated and is illite-dominated clay [54]. The biochar was produced from wheat straw in a laboratory muffle furnace under limited oxygen conditions at 500 °C, as described by [55]. Compost was produced from agricultural waste material and plant leaves as described by [56]. The biochar and compost were dried at 70 °C in an oven for 3 days, ground to a fine powder manually with a grinder and roller, passed through a 200 µm sieve and stored for use. 

### 3.2. Analytical Methods

Before the experiment, the soil was analyzed for soluble cations and anions, texture, sodium adsorption ratio (SAR) total nitrogen (N), phosphorus (P), potassium (K), cation exchange capacity and CaCO_3_ following the procedures as stated [57]. Manganese (Mn), iron (Fe) and zinc (Zn) contents in soil were determined by using the aqua regia method (HNO_3_: HCl; 1:3) [58]. The pH and EC of soil and amendments were measured by a suspension method (1:10 [w/v] and 1:20 [w/v] solid–distilled water ratio), respectively after shaking for 90 min in deionized water on a mechanical shaker [59] using a pH (JENCO Model-671P) and conductivity meter (HANNA HI8033), respectively. Total N was determined by the Kjeldahl method [60]. The concentrations of Mn, Fe, Zn, K and P were determined from compost and biochar samples by digesting them in sulphuric acid (H_2_SO_4_) and hydrogen peroxide (H_2_O_2_) [59]. Next, P was determined by a spectrophotometer, K was determined by a flame photometer and Fe, Zn and Mn were determined by an atomic absorption spectrophotometer.

### 3.3. Pesticide and Chemicals

Analytical grade CP (99.5%) was obtained from Dr. Ehrenstorfer GmbH Wesel, Nordrhein-Westfalen, Germany. Ali Akbar Enterprises, Pvt. Ltd. Lahore, Pakistan supplied the technical grade CP (98% pure). The analytical grade acetone and n-hexane used were purchased from Merck Darmstadt, Germany. The Florisil and sodium sulphate dehydrates used in the cleanup process and extractions were purchased from Sigma-Aldrich (Sydney, Australia).

### 3.4. Plant Growth Experiment

Maize (*Zea mays* L.) grown in sampled soil (sandy clay loam texture) was used as a test crop in this study. The experiment was conducted in a wire house using plastic pots to allow no leaching of water and pesticide. Before filling the pots, soil and amendments were thoroughly mixed to achieve 0.25% and 0.50% (*w/w*) of biochar and compost on a soil dry weight basis. Each pot was filled with 2.5 kg of soil (with or without amendment). The soil was contaminated with CP solution in acetone, resulting in the spiked concentration of 100 and 200 mg kg^−1^ of CP. The treatment combinations have been explained in Table 2. The experiment was conducted using a completely randomized design with three replications of each treatment, and a total of 33 pots were kept. The pots were agitated on an orbital shaker for 24 h to ensure complete mixing of soil and pesticide solution. When all carrier acetone was evaporated after another 2 days, the deionized water was added to adjust the moisture contents at 50% of water holding capacity. Four maize seeds were sown in each pot. The fertilizers were applied in recommended doses using urea (CO (NH_2_)_2_), diammonium phosphate (NH_4_)_2_HPO_4_ and sulphate of potash (K_2_SO_4_) at 120–90–60 kg NPK ha^−1^. All the P and K were applied at the time of sowing while N was applied in three splits. The plants were harvested after 60 days. The above-ground parts of maize were cut on soil surface [61]. The maize roots were carefully removed from the soil [62]. The growth parameters of shoots and roots were determined. A small portion (5 g) of soil was removed from each pot after thorough mixing for CP residue determination in postharvest soil. The shoots and roots were thoroughly washed with deionized water to remove soil particles and were air dried at room temperature in the laboratory.

### 3.5. Residue Extraction and Cleanup

The extraction and cleanup of CP from plant and soil samples (shoots and roots) was conducted as per the procedure stated in [11]. The plant sample (2.5 g) was ground in a pestle and mortar with 10 g of sodium sulphate dehydrates. The extraction of the mixture was carried out with 15 ml of n-hexane and acetone (1:1 *v/v*). The extraction procedure was as follows: vortex mixing of mixture for one minute, ultrasonication for two hours, shaking on an orbital shaker for 12 hours and centrifugation for 15 minutes at 1300 RPM for phase separation. The supernatant was removed following centrifugation and was dried under N_2_ gas. The residues were redissolved in 1 ml acetone. The extracts were then further purified by Florisil cleanup process. First of all, the column was washed with n-hexane (5 mL) and the extract was passed through Florisil. After this, the column was washed again to wash out CP sorbed by the Florisil by further using 5 mL of hexane/dichloromethane (1:1, *v/v*). The extract was then dried under N_2_, and dissolved in 1 mL acetone to determine CP by GC-MS. A recovery experiment was carried out with the fortification of plant materials with CP ranging from 1 to 10 mg kg^−1^. The recovery ranged from 75% to 90%. For extraction of soil samples, 10 mL of n-hexane and acetone (1:1 *v/v*) was added to 1 g of soil and extraction was conducted with 10 mL of n-hexane and acetone using the above-mentioned procedure. For the recovery experiment, the soil samples were spiked with CP 1–50 mg kg^−1^. The recoveries for soil samples ranged from 80–90%.

### 3.6. Residue Analysis

The analysis for CP concentration in plant and soil samples was carried out on GC-MS (Shimadzu QP-2010) Kyoto, Japan. The instrumental conditions were as follows: Injection mode was splitless with the sampling time of 1 min. The injection temperature was 220 °C. The carrier gas was 99.9% Helium. The flow rate of helium gas was 1.70 mL min^−1^. The temperature of the oven was 50 °C (1 min) ramping to 180 °C at 20 °C min^−1^, to 190 °C at 10 °C to 240 °C at 3 °C min^−1^, to 300 °C at 10 °C min^−1^ and then held for 6 min. Total program time was 37.17 min. The MS conditions were as follows: Solvent cut time was 5 min. The ion source temperature was 200 °C. MS interface temperature was 280 °C. The detection of CP was achieved using selected ion mode. For CP the mass fragments monitored were m/z 197, 199 and 314. 

### 3.7. Extraction and Determination of Enzyme Activities

Enzyme extract was prepared by the procedure stated in [11], taking 0.5 g of plant samples with liquid N_2_. The plant material was crushed with a pestle and mortar (which was kept cold before and during crushing to prevent heating). After this, 15% glycerol, 1% Triton X-100, 1 mM EDTA and 2 mL of 100 mM potassium phosphate buffer (pH 7.8) were added to make a mixture. The centrifugation of the mixture was conducted for 15 min at 4 °C and 15,000 rpm. The supernatant was removed and stored at −20 °C.

The procedure stated by [63] was adopted for superoxide dismutase (SOD EC. 1.15.1.1) determination by using a UV-visible spectrophotometer at 560 nm. The indication of SOD was the inhibition of photochemical reduction of nitro blue tetrazolum (NBT). To determine SOD activity, 500 μL of 75 mM EDTA, 950 μL of 50 mM phosphate buffer pH (7.8), 500 μL of 13 mM Methionine, 1.3 μM Riboflavin and 1 mL of 50 μM NBT were used as a reaction mixture in 50 μL of enzyme extract. One unit of SOD was defined as the amount of enzyme required to cause 50% inhibition of the NBT reduction rate compared to blanks (tubes in which enzyme extract was not added) at 560 nm.

Catalase (CAT EC. 1.11.1.6) activity was assayed using the method described by [64], by quantifying titanium–hydro complex formation by estimating residual hydrogen peroxide. Next, 0.2 mL enzyme extract was taken and 6 mM H_2_O_2_, and 0.1 mM phosphate buffer (pH 7.0) was added to form 3 mL of reaction mixture. Next, 2 mL of titanium reagent was added to stop the reaction. This resulted in the formation of yellow titanium–hydroperoxide complex with residual hydrogen peroxide. After 30 min the centrifugation of the aliquot was performed for 10 min at 10,000 g. The supernatant was removed and ran on a spectrophotometer to record absorbance at 410 nm, and catalase activity was measured by the decline in absorbance (at 410 nm) due to the extinction of H_2_O_2_ and expressed as µmoles min^−1^ mg^−1^ protein. 

Peroxidase (POX EC. 1.11.1.7) activity was determined using the procedure stated by [65], by estimating the tetraguaiacol formation resulting in the increment of optical density. The enzyme extract (0.1 mL) was first diluted 10 times. After this, 0.15 M phosphate buffer (pH 6.1), 2 mM H_2_O_2_ and 16 mM guaiacol were added to form 3 mL of reaction mixture. After centrifugation of the aliquot, the absorbance of the supernatant was quantified using a spectrophotometer at 470 nm, and calculation of enzyme activity was conducted as per its extinction coefficient of 26.6 mM^−1^ cm^−1^. 

### 3.8. Statistical Analysis

The experiment was conducted using a completely randomized design. Statistical analysis was performed via one-way factorial analysis of variance (ANOVA). The means were compared by applying a least significant difference (LSD) test at *p* < 0.05 for critical differences between treatments, using Statistics Software version 8.1.1. Data with less than 5% (*p* < 0.05) probabilities were considered statistically significant [66].

## 4. Conclusion

CP significantly reduced maize growth. Maize plants showed increased residual concentration of CP in both shoots and roots with increasing level of CP. Maize plants induced variations in antioxidant enzyme activities in response to CP stress. Application of both biochar and compost amendments alleviated the adverse effects of CP in all studied parameters, as manifested by the profound improvement in maize fresh biomass, recovered antioxidant enzyme activities, and decreased residual CP concentration in both roots and shoots of maize. However, biochar at the 0.50% level was found to be more effective in reducing uptake of CP by maize plants compared with compost. More CP was dissipated in compost-amended treatments compared with biochar. This study is of practical significance and emphasizes that both biochar and compost amendments could effectively be used to minimize CP entry into agricultural produce by reducing its bioavailability to maize plants, and this could be implied for other crop species. However, further research is warranted to delineate the mechanism of immobilization of CP by biochar and compost amendments. Present results should be explored on a field level, with different soils having different histories of CP contamination.

## Figures and Tables

**Table 1:** Selected physicochemical properties of the soil and amendments.

Characteristic	Soil	Compost	Biochar
Texture	Sandy clay loam	--	--
Sand (%)	56.4 ± 1.04	--	--
Silt (%)	18.9 ± 0.98	--	--
Clay (%)	24.7 ± 1.01	--	--
pH_w(1:10)_	7.44 ± 0.10	6.25 ± 0.09	7.89 ± 0.08
EC_w(1:10)_ (dS m^−1^)	3.21 ± 0.08	3.10 ± 0.15	4.01 ± 0.08
TSS (mmol_c_ L^−1^)	32 ± 0.20	--	--
CaCO_3_	4.91	--	--
Total organic carbon (%)	0.87± 0.03	35.36 ± 1.32	43.80 ± 1.65
Specific surface area (m^2^ g^−1^)	--	31.37 ± 0.04	94.83± 0.09
Pore width (nm)	--	21 ± 1.32	15.0 ± 0.91
Pore volume (cm^3^ g^−1^)	--	0.0035 ± 0.0001	0.09 ± 0.0001
CEC cmol_c_kg^−1^	5.2 ± 0.87	107.5 ± 4.34	85 ± 3.94
Fe (mg kg^−1^)	5.5 ± 0.91	755.6 ± 87	154.6 ± 11
Mn (mg kg^−1^)	0.51 ± 0.001	103.35 ± 9	395.62 ± 13
Zn (mg kg^−1^)	0.91 ± 0.01	130.3 ± 11	78.3 ± 8
Total N (%)	0.03 ± 0.001	1.59 ± 0.02	0.83 ± 0.01
Available P (%)	0.0007 ± 0.0001	1.30 ± 0.01	0.20 ± 0.001
Extractable K (%)	0.014 ± 0.001	2.59 ± 0.03	1.06 ± 0.01

Values are presented as means ± standard error of three replicates, EC: Electrical conductivity, TSS: Total soluble salts, CEC: Cation exchange capacity.

**Table 2:** Treatment description and their abbreviations used in the study.

Treatment	Abbreviations
Control	CP_0_B_0_C_0_
CP 100 mg kg^−1^	CP_100_
CP 200 mg kg^−1^	CP_200_
CP100 mg kg^−1^ + compost 0.25%	CP_100_C_0.25_
CP200 mg kg^−1^ + compost 0.25%	CP_200_C_0.25_
CP100 mg kg^−1^ + compost 0.50%	CP_100_C_0.50_
CP200 mg kg^−1^ + compost 0.50%	CP_200_C_0.50_
CP100 mg kg^−1^ + biochar 0.25%	CP_100_B_0.25_
CP200 mg kg^−1^ + biochar 0.25%	CP_200_B_0.25_
CP100 mg kg^−1^ + biochar 0.50%	CP_100_B_0.50_
CP200 mg kg^−1^ + biochar 0.50%	CP_200_B_0.50_

## Data Availability

Not applicable.

## References

[1] Pelosi C., Bertrand C., Daniele G., Coeurdassier M., Benoit P., Nelieu S., Fritsch C. (2021). Residues of currently used pesticides in soils and earthworms: A silent threat?. Agric. Ecosyst. Environ..

[2] Liu D.B., Chen W.W., Wei J.H., Li X.B., Wang Z., Jiang X.Y. (2012). A highly sensitive, dual-readout assay based on gold nanoparticles for organophosphorus and carbamate pesticides. Anal. Chem..

[3] Howard P.H., Gray D.A., Sage G.W., Jarvis W.F. (1990). Handbook of Environmental Fate and Exposure 311 Data for Organic Chemicals. Volume II: Solvents.

[4] Rajmohan K., Chandrasekaran R., Varjani S. (2020). A review on occurrence of pesticides in environment and current technologies for their remediation and management. Indian J. Microbiol..

[5] Ahad K., Anwar T., Ahmad I., Mohammad A., Tahir S., Aziz S., Baloch U.K. (2000). Determination of insecticides residues in ground water of Mardan Division, NWFP. Pakistan: A case study. Water SA.

[6] Parveen Z., Khuhro M.I., Rafiq N., Kausar N. (2004). Evaluation of multiple pesticide residues in apple and citrus fruits, 1999–2001. Bull. Environ. Contam. Toxicol..

[7] Muhammad F., Akhtar M., Rahman Z.U., Farooq H.U., Khaliq T., Anwar M.I. (2010). Multi-residue determination of pesticides in the meat of cattle in Faisalabad Pakistan. Egypt Acad. J. Biol. Sci..

[8] Ismail M., Ali R., Shahid M., Khan M.A., Zubair M., Ali T., Khan Q.M. (2017). Genotoxic and hematological effects of chlorpyrifos exposure on freshwater fish Labeorohita. Drug Chem. Toxicol..

[9] Duntas L.H., Stathatos N. (2015). Toxic chemicals and thyroid function: Hard facts and lateral thinking. Rev. Endocr. Metab. Dis..

[10] Wang C., Zhang Q. (2017). Exogenous salicylic acid alleviates the toxicity of chlorpyrifos in wheat plants (*Triticum aestivum* L.). Ecotoxicol. Environ. Saf..

[11] Yu X.Y., Ying G., Kookana R.S. (2009). Reduced plant uptake of pesticides with biochar additions to soil. Chemosphere.

[12] Zhang Z.Y., Shan W.L., Song W.C., Gong Y., Liu X.J. (2011). Phytotoxicity and uptake of chlorpyrifos in cabbage. Environ. Chem. Lett..

[13] Gvozdenac S., Indic D., Vukovic S. (2013). Phytotoxicity of Chlorpyrifos to White Mustard (*Sinapis alba* L.) and Maize (*Zea mays L.*): Potential Indicators of Insecticide Presence in Water. Pestic. Phytomed..

[14] Dubey P., Mishra A.K., Shukla P., Singh A.K. (2015). Differential sensitivity of barley (*Hordeum vulgare* L.) to chlorpyrifos and propiconazole: Morphology, cytogenetic assay and photosynthetic pigments. Pestic. Biochem. Phys..

[15] Tahir M., Hassan A.U., Maqbool S., Barber B., Koskinen W.C., Xinhua P.E.N.G., Mulla D.J. (2016). Sorption and leaching potential of isoproturon and atrazine in low organic carbon soil of Pakistan under a wheat-maize rotation. Pedosphere.

[16] Tahir M., Hassan A.U., Zahir Z.A., Ur-Rehman K. (2012). Modeling the water retention capacity and hydraulic properties of a manure-amended loam soil and its effect on wheat and maize yield. Int. J. Agric. Biol..

[17] Saleem M., Ali S., Rehman M., Rana M., Rizwan M., Kamran M., Imran M., Riaz M., Hussein M., Elkelish A. (2020). Influence of phosphorus on copper phytoextraction via modulating cellular organelles in two jute (*Corchorus capsularis L.*) varieties grown in a copper mining soil of Hubei Province, China. Chemosphere.

[18] Javed M.T., Saleem M.H., Aslam S., Rehman M., Iqbal N., Begum R., Ali S., Alsahli A.A., Alyemeni M.N., Wijaya L. (2020). Elucidating silicon-mediated distinct morpho-physio-biochemical attributes and organic acid exudation patterns of cadmium stressed Ajwain (*Trachyspermum ammi L.*). Plant Physiology and Biochemistry.

[19] Iqbal S., Christian T., Haroon Z.K., Hafiz M.R.J., Muhammad A., Muhammad S. (2017). Maximizing maize quality, productivity and profitability through a combined use of compost and nitrogen fertilizer in a semi-arid environment in Pakistan. Nutr. Cycl. Agroecosyst..

[20] Agegnehu G., Bass A.M., Nelson P.N., Bird M.I. (2016). Benefits of biochar, compost and biochar–compost for soil quality, maize yield and greenhouse gas emissions in a tropical agricultural soil. Sci. Total Environ..

[21] Mahmood F., Khan I., Ashraf U., Shahzad T., Hussain S., Shahid M., Ullah S. (2017). Effects of organic and inorganic manures on maize and their residual impact on soil physico-chemical properties. J. Soil Sci. Plant Nutr..

[22] Cederlund H., Börjesson E., Stenström J. (2017). Effects of a wood-based biochar on the leaching of pesticides chlorpyrifos, diuron, glyphosate and MCPA. J. Environ. Manag..

[23] Burgess R.M., Perron M.M., Friedman C.L., Suuberg E.M., Pennell K.G., Cantwell M.G., Ryba S.A. (2009). Evaluation of the effects of coal fly ash amendments on the toxicity of a contaminated marine sediment. Environ. Toxicol. Chem..

[24] Li Y., He J., Qi H., Li H., Boyd S.A., Zhang W. (2020). Impact of biochar amendment on the uptake, fate and bioavailability of pharmaceuticals in soil-radish systems. J. Hazard. Mater..

[25] Hilber I., Wyss G.S., Mader P., Bucheli T.D., Meier I., Vogt L., Schulin R. (2009). Influence of activated charcoal amendment to contaminated soil on dieldrin and nutrient uptake by cucumbers. Environ. Pollut..

[26] Yang Y.N., Sheng G.Y. (2003). Enhanced pesticide sorption by soils containing particulate matter from crop residue burns. Environ. Sci. Technol..

[27] Parween T., Jan S., Fatma T. (2011). Alteration in nitrogen metabolism and plant growth during different developmental stages of green gram (*Vigna radiata* L.) in response to chlorpyrifos. Acta Physiol. Plant..

[28] Dubey K.K., Fulekar M.H. (2011). Effect of pesticides on the seed germination of Cenchrus setigerus and Pennisetum pedicellatum as monocropping and co-cropping system: Implications for rhizospheric bioremediation. Rom. Biotechnol. Lett..

[29] Yang X., Guoying G., Anpeng P., Zhao L.J.L., Zhang L.J., Yuan P., He H.P. (2010). Influence of Biochars on Plant Uptake and Dissipation of Two Pesticides in an Agricultural Soil. J. Agric. Food Chem..

[30] Tang X., Huang W., Guo J., Yang Y., Tao R., Xu F. (2017). Use of Fe-impregnated Biochar to Efficiently Sorb Chlorpyrifos, reduce uptake by *Allium fistulosum* L. and Enhance Microbial Community diversity. J. Agric. Food Chem..

[31] Antonious G.F. (2015). Decontamination of Pesticide Residues for Sustainable Agriculture. JSM. Environ. Sci. Ecol..

[32] Lehmann J., Gaunt J., Rondon M. (2006). Bio-char sequestration in terrestrial ecosystems—A review. Mitig. Adapt. Strat. Glob. Chang..

[33] Song H., Yin X., Chen G.F., Yang H. (2007). Biological responses of wheat (*Triticum aestivum* L.) plants to the herbicide chlorotoluron in soils. Chemosphere.

[34] Chen S., Chen M., Wang Z., Qiu W., Wang J., Shen Y., Ge S. (2016). Toxicological effects of chlorpyrifos on growth, enzyme activity and chlorophyll a synthesis of freshwater microalgae. Environ. Toxicol. Pharmacol..

[35] Parween T.A., Jan S., Fatma T. (2012). Evaluation of oxidative stress in *Vigna radiata* L. in response to chlorpyrifos. Int. J. Environ. Sci. Technol..

[36] Bashir F., Siddiqi T.O., Iqbal M. (2007). The anti-oxidative response system in *Glycine max* L.) Merr. exposed to deltamethrin, a synthetic pyrethroid insecticide. Environ. Pollut..

[37] Jianga L., Maa L., Suia Y., Hna S.Q., Wua Z.Y., Fenga Y.X., Yanga H. (2010). Effect of manure compost on the herbicide prometryne bioavailability to wheat plants. J. Hazard. Mater..

[38] Cakmak I. (2000). Possible roles of zinc in protecting plant cells from damage by reactive oxygen species. New Phytol..

[39] Corpas F.J., Barroso J.B. (2017). Lead-induced stress, which triggers the production of nitric oxide (NO) and superoxide anion (O^2−^) in Arabidopsis peroxisomes, affects catalase activity. Nitric Oxide.

[40] Azcon R., del M., Peralvarez C., Biro B., Roldan A., Ruiz-Lozano J.M. (2009). Antioxidant activities and metal acquisition in mycorrhizal plants growing in a heavy-metal multicontaminated soil amended with treated ligno cellulosic agrowaste. Appl. Soil Ecol..

[41] Fang H., Yun-long Y., Xiao Wang M.S., Xiao-mao W., Jing-quan Y. (2006). Dissipation of chlorpyrifos in pakchoi-vegetated soil in a greenhouse. J. Environ. Sci..

[42] Mutua G.K., Ngigi A.N., Getenga Z.M. (2015). Chlorpyrifos Degradation in Soils with Different Treatment Regimes within Nzoia River Drainage Basin, Kenya. Bull. Environ. Contam. Toxicol..

[43] Garcia-Jaramillo M., Redondo-Gómez S., Barcia-Piedras J.M., Aguilar M., Jurado V., Hermosín M.C., Cox L. (2016). Dissipation and effects of tricyclazole on soil microbial communities and rice growth as affected by amendment with alperujo compost. Sci. Total Environ..

[44] Kravvariti K., Tsiropoulos N.G., Karpouzas D.G. (2010). Degradation and adsorption of terbuthylazine and chlorpyrifos in biobedbiomixtures from composted cotton crop residues. Pest Manag. Sci..

[45] Lopez-Pin eiro A., Cabrera D., Albarran A., Pena D. (2011). Influence of two-phase olive mill waste application to soil on terbuthylazine behavior and persistence under controlled and field conditions. J. Soils Sediments.

[46] Copaja S.V., Vergara R., Bravo H.R. (2014). Bioavailability of Chlorpyrifos in Wheat Plants (*Triticum aestivun* L.). Agric. Sci..

[47] Hamaker J.W., Thompson J.M., Hamaker J.W., Goring C.A.I. (1972). Adsorption of organic chemicals in the soil environment. Organic Chemicals in the Soil Environment.

[48] Moyo F., Tandlich R., Wilhelmi B.S., Balaz S. (2014). Sorption of hydrophobic organic compounds on natural sorbents and organoclays from aqueous and non-aqueous solutions: A mini-review. Int. J. Environ. Res. Public Health.

[49] Deng H., Feng D., He J.X., Li F.Z., Yu H.M., Ge C.J. (2017). Influence of biochar amendments to soil on the mobility of atrazine using sorption-desorption and soil thin-layer chromatography. Ecol. Eng..

[50] Zbytniewski R., Buszewski B. (2002). Sorption of pesticides in soil and compost. Pol. J. Environ. Stud..

[51] Medina J., Monreal C., Chabot D., Meier S., Gonzalez M.E., Morales E., Cornejo P. (2017). Microscopic and spectroscopic characterization of humic substances from a compost amended copper contaminated soil: Main features and their potential effects on Cu immobilization. Environ. Sci. Pollut. Res..

[52] Zhang X., Shen Y., Yu X., Liu X. (2012). Dissipation of chlorpyrifos and residue analysis in rice, soil and water under paddy field conditions. Ecotoxicol. Environ. Saf..

[53] Khorram M.S., Zheng Y., Lin D., Zhang Q., Fang H., Yu Y. (2016). Dissipation of fomesafen in biochar-amended soil and its availability to corn (*Zea mays* L.) and earthworm (*Eisenia fetida* L.). J. Soils Sediments.

[54] Murtaza B., Murtaza G., Saqib M., Khaliq A. (2014). Efficiency of nitrogen use in rice-wheat cropping system in salt-affected soils with contrasting texture. Pak. J. Agric. Sci..

[55] Sanchez M.E., Lindao E., Margaleff D., Martınez O., Moran A. (2009). Pyrolysis of agricultural residues from rape and sunflowers: Production and characterization of bio-fuels and biochar soil management. J. Anal. Appl. Pyrol..

[56] Ahmad R., Shahzad S.M., Khalid A., Arshad M., Mahmood M.H. (2007). Growth and yield response of wheat (*Triticum aestivum* L.) and maize (*Zea mays* L.) to nitrogen and L-tryptophan enriched compost. Pak. J. Bot..

[57] Salinity Lab. Staff US (1954). Diagnosis and Improvement of Saline and Alkali Soils.

[58] Haynes R.J., Murtaza G., Naidu R. (2009). Inorganic and organic constituents and contaminants of biosolids: Implications for land application. Adv. Agron..

[59] Wolf B. (1983). The comprehensive system of leaf analysis and its use for diagnosing crop nutrient status. Commun. Soil Sci. Plant Anal..

[60] Barbano D.M., Clark J.L., Dunham C.E., Flemin R.J. (1990). Kjeldahl method for determination of total nitrogen content of milk: Collaborative study. J. Assoc. Off. Anal. Chem..

[61] Al-Wabel M.I., Usman A.R., El-Naggar A.H., Aly A.A., Ibrahim H.M., Elmaghraby S., Al-Omran A. (2015). Conocarpus biochar as a soil amendment for reducing heavy metal availability and uptake by maize plants. Saudi J. Biol. Sci..

[62] Aziz H., Sabir M., Ahmad H.R., Aziz T., Zia-ur-Rehman M., Hakeem K.R., Ozturk M. (2015). Alleviating effect of calcium on nickel toxicity in rice. CLEAN-Soil Air Water.

[63] Giannopolitis C.N., Reis S.K. (1997). Superoxide dismutase occurrence in higher plants. Plant Physiol..

[64] Teranishi Y., Tanaka A., Osumi M., Fukui S. (1974). Catalase activity of hydrocarbon utilizing candida yeast. Agric. Biol. Chem..

[65] Castillo F.I., Penel I., Greppin H. (1984). Peroxidase release induced by ozone in Sedum album leaves. Plant Physiol..

[66] Steel R.G.D., Torrie J.H., Dickey D.A. (1997). Principles and Procedures of Statistics.

